# Cardioprotective Glucose-Lowering Agents and Dementia Risk: A Systematic Review and Meta-Analysis

**DOI:** 10.1001/jamaneurol.2025.0360

**Published:** 2025-04-07

**Authors:** Allie Seminer, Alfredi Mulihano, Clare O’Brien, Finn Krewer, Maria Costello, Conor Judge, Martin O’Donnell, Catriona Reddin

**Affiliations:** 1 HRB Clinical Research Facility, University of Galway, Galway, Ireland; 2 University Hospital Galway, Galway, Ireland; 3 Wellcome Trust-HRB, Irish Clinical Academic Training, Dublin, Ireland

## Abstract

**Importance:**

Although diabetes is a risk factor for dementia, the effect of glucose-lowering therapy for prevention of incident dementia is uncertain.

**Objective:**

To determine whether cardioprotective glucose-lowering therapy (sodium-glucose cotransporter-2 inhibitors [SGLT2is], glucagon-like peptide-1 receptor agonists [GLP-1RAs], metformin, and pioglitazone), compared with controls, was associated with a reduction in risk of dementia or cognitive impairment, and among primary dementia subtypes.

**Data Sources:**

The PubMed and Embase databases were searched for studies published from inception of the database to July 11, 2024.

**Study Selection:**

Randomized clinical trials comparing cardioprotective glucose-lowering therapy with controls that reported dementia or change in cognitive scores. Cardioprotective glucose-lowering therapies were defined as drug classes recommended by guidelines for reduction of cardiovascular events, based on evidence from phase III randomized clinical trials. Inclusion criteria were assessed independently and inconsistencies were resolved by consensus.

**Data Extraction and Synthesis:**

Data were screened and extracted independently by 2 authors adhering to the PRISMA guidelines in August 2024. Random-effects meta-analysis models were used to estimate a pooled treatment effect.

**Main Outcomes and Measures:**

The primary outcome measure was dementia or cognitive impairment. The secondary outcomes were primary dementia subtypes, including vascular and Alzheimer dementia, and change in cognitive scores.

**Results:**

Twenty-six randomized clinical trials were eligible for inclusion (N = 164 531 participants), of which 23 trials (n = 160 191 participants) reported the incidence of dementia or cognitive impairment, including 12 trials evaluating SGLT2is, 10 trials evaluating GLP-1RAs, and 1 trial evaluating pioglitazone (no trials of metformin were identified). The mean (SD) age of trial participants was 64.4 (3.5) years and 57 470 (34.9%) were women. Overall, cardioprotective glucose-lowering therapy was not significantly associated with a reduction in cognitive impairment or dementia (odds ratio [OR], 0.83 [95% CI, 0.60-1.14]). Among drug classes, GLP-1RAs were associated with a statistically significant reduction in dementia (OR, 0.55 [95% CI, 0.35-0.86]), but not SGLT2is (OR, 1.20 [95% CI, 0.67-2.17]; *P* value for heterogeneity = .04).

**Conclusions and Relevance:**

While cardioprotective glucose-lowering therapies were not associated with an overall reduction in all-cause dementia, this meta-analysis of randomized clinical trials found that glucose lowering with GLP-1RAs was associated with a statistically significant reduction in all-cause dementia.

## Key Points

**Question:**

Are cardioprotective glucose-lowering agents associated with reduced risk of dementia or cognitive impairment?

**Findings:**

This systematic review and meta-analysis of 26 randomized clinical trials (N = 164 531) found no significant association between cardioprotective glucose-lowering therapy and reductions in cognitive impairment or dementia. Among drug classes, glucagon-like peptide-1 receptor agonists (GLP-1RAs) were associated with a statistically significant reduction in dementia, but not sodium-glucose cotransporter-2 inhibitors (SGLT2is).

**Meaning:**

In this meta-analysis of randomized clinical trials, glucose-lowering therapy with GLP1-RAs, but not SGLT2is, was associated with a statistically significant reduction in dementia or cognitive impairment.

## Teaser

This meta-analysis examines the association of cardioprotective glucose-lowering agents, including glucagon-like peptide-1 receptor agonists and sodium-glucose cotransporter-2 inhibitors, with reductions in dementia and cognitive impairment.

## Introduction

Dementia is a leading cause of disability globally, and is projected to affect approximately 75 million people by 2030.^1,2^ Diabetes is a risk factor for dementia, estimated to account for approximately 5% of the population-attributable fraction of all dementia.^3,4^ Diabetes is also a risk factor for ischemic stroke,^5^ which may mediate an association with vascular dementia. Identification of population-level interventions, such as tailored management of common risk factors (eg, diabetes), may reduce the global burden of dementia. However, there is a lack of robust evidence to support the efficacy of glucose-lowering therapies in reducing the risk of dementia, which may relate to variations in cardioprotective efficacy among glucose-lowering therapies.

In patients with type 2 diabetes and cardiovascular disease or risk factors, guidelines recommend use of sodium-glucose cotransporter-2 inhibitor (SGLT2i) and glucagon-like peptide-1 receptor agonist (GLP-1RA) drug classes as first-line treatments, based on findings from phase III randomized clinical trials reporting a significant reduction in cardiovascular events, and metformin and pioglitazone are recommended as second-line treatments.^6,7^ Observational evidence suggests that certain glucose-lowering drug classes, such as SGLT2is and GLP-1RAs, may have a neuroprotective effect.^8,9^ However, to date, the association of cardioprotective classes of glucose-lowering therapies with risk of dementia has been inconsistent, prompting the need for an updated meta-analysis of randomized clinical trials.^10,11^

The aim of this meta-analysis was to determine whether cardioprotective glucose-lowering therapy, compared with controls, was associated with a reduction in risk of dementia or cognitive impairment, and among primary dementia subtypes.

## Methods

We performed a systematic review and meta-analysis, adhering to the Cochrane Collaboration guidelines, and reported our findings according to the Preferred Reporting Items for Systematic Reviews and Meta-Analyses (PRISMA) guidelines.^12,13^ The meta-analysis was registered with the International Prospective Register of Systematic Reviews (PROSPERO; CRD42024557562). The data that support the findings of this study are available from the corresponding author upon reasonable request.

### Data Sources and Search Strategy

We systematically searched the PubMed and Embase databases for articles published from inception of the database to June 11, 2024. The included search terms are detailed in the eMethods in Supplement 1. The reference list of studies selected for inclusion and published systematic reviews of glucose-lowering therapy were also screened for studies that met our inclusion criteria. Two reviewers (A.S. and A.M.) independently screened titles and abstracts. Full texts were sourced for relevant articles. To ascertain if cognitive outcomes were reported, the full text, supplementary appendix, and ClinicalTrials.gov record were reviewed for each study that met inclusion criteria related to the population, intervention, and comparator. Inclusion criteria were assessed independently and inconsistencies were resolved by consensus.

### Eligibility Criteria

Studies were considered eligible if they (1) were randomized clinical trials; (2) included adults older than 18 years; (3) evaluated cardioprotective glucose-lowering therapy based on guideline recommendations and findings from phase III randomized clinical trials compared with controls; (4) reported dementia, cognitive impairment, and/or change in cognitive score; and (5) had a 6-month minimum follow-up duration. Eligible glucose-lowering interventions included the following drug classes and agents: SGLT2is, GLP-1RAs, metformin, and pioglitazone. These drug classes are recommended as glucose-lowering therapy for individuals with cardiovascular disease or risk factors^6,7^ on the basis of randomized clinical trial evidence demonstrating a reduction in cardiovascular events (SGLT2is and GLP-1RAs)^14,15,16^ or suggesting cardiovascular benefit (metformin and pioglitazone).^17,18^ The following drug classes were excluded due to lack of current evidence of efficacy in cardiovascular outcome trials: dipeptidyl peptidase-4 inhibitors,^19^ sulfonylureas,^20^ and insulin.^21,22^ Controls were defined as placebo, usual care, or no glucose-lowering therapy. Trials evaluating the effect of glucose-lowering therapy in a population with a prior diagnosis of cognitive impairment or dementia were excluded.

### Data Extraction

Data were abstracted independently by 2 authors (A.S. and A.M.) using a standardized data collection form. For each study, we abstracted the title, year of publication, glucose-lowering drug (class, drug name, and dose), control, design (open label or placebo controlled), intervention and control numbers, definition of dementia used, all-cause dementia, dementia subtypes, and change in cognitive score. We reported outcomes from the point of longest available follow-up. Data were compared for inconsistencies and merged into a prefinal dataset, which was checked independently by a third reviewer (C.R.).

### Outcomes

The primary outcome of this meta-analysis was dementia or cognitive impairment on follow-up. We used a hierarchical approach in which we included trials that reported incident dementia, or a composite of dementia or cognitive impairment (if dementia alone was not reported), on follow-up.^23^ Secondary outcomes included dementia subtypes, including vascular dementia and Alzheimer dementia, and change in cognitive score.

### Risk-of-Bias Assessment

The methodological quality of eligible trials was assessed using the Cochrane Risk of Bias 2 tool.^24^ Two independent reviewers (A.M. and C.O.) performed risk-of-bias assessments, and disagreements were resolved by a third reviewer (C.R.). If 1 of the domains was rated as high risk, the study was considered at a high risk of bias.

### Data Synthesis and Analysis

A descriptive analysis of included trials and definitions of primary outcomes are reported in the Table. We calculated the odds ratio (OR) and 95% CIs for each outcome of interest from individual studies. Weighted pooled treatment effects were calculated overall and individually for individual drug classes, using restricted maximum likelihood (REML) estimation to fit a random-effects meta-analysis model. REML estimation was chosen because it has been shown to be less biased than the DerSimonian-Laird estimator.^50,51^ Absolute risk reductions (ARRs) and 95% CIs were calculated for each study; the Mantel-Haenszel method was used to obtain a pooled estimate of the risk difference. The variability across studies due to heterogeneity was investigated using forest plots and *I^2^* statistics. Differences in cognitive scores were not meta-analyzed due to lack of data and heterogeneity of score used. Publication bias was assessed using a funnel plot.

**Table.** Characteristics of Included Trials

Source	No. of participants	Trial design	Study population	Mean age, y	No. of female participants (%)	Group		Follow-up, mo	Primary outcome definition
						Intervention	Control		
**SGLT2is**									
EMPA-REG OUTCOME,^25^ 2015	7020	Double-blind, placebo-controlled RCT	Aged ≥18 y with T2D, BMI ≤45, eGFR ≥30 mL/min/1.73 m^2^ of body surface area, and established CVD	63.2	2004 (28.5)	Empagliflozin	Placebo	37.3	Reported as adverse event (Alzheimer dementia)
CANVAS-R,^26^ 2017	5812	Quadruple-blind, placebo-controlled RCT	Aged ≥30 y with T2D, HbA_1c_ ≥7% to ≤10.5%, eGFR ≥30 mL/min/1.73 m^2^, and CV risk factors or previous CV event, or ≥50 y with risk of CV event	64.0	2164 (37.2)	Canagliflozin	Placebo	24.8	Reported as adverse event (cognitive disorder)
CANVAS,^26^ 2017	4330	Quadruple-blind, placebo-controlled RCT	Aged ≥30 y with T2D, HbA_1c_ ≥7%, CV risk factors or previous CV event, and eGFR ≥30 mL/min/1.73 m^2^	62.4	1469 (33.9)	Canagliflozin	Placebo	73.2	Reported as adverse event (vascular cognitive impairment)
CREDENCE,^27^ 2019	4401	Double-blind, placebo-controlled RCT	Aged ≥30 y with T2D, HbA_1c_ 6.5%-12%, eGFR 30 to <90 mL/min/1.73 m^2^, and albuminuria	63.1	1494 (33.9)	Canagliflozin	Placebo	31.44	Reported as adverse event (dementia)
DAPA-HF,^28^ 2019	4744	Quadruple-blind, placebo-controlled RCT	Aged ≥18 y with a diagnosis of symptomatic HFrEF for at least 2 mo, LVEF ≤40%, and elevated NT-proBNP levels	66.4	1109 (23.3)	Dapagliflozin	Placebo	18.2	Reported as adverse event (vascular dementia)
DECLARE-TIMI 58,^29^ 2019	17 160	Quadruple-blind, placebo-controlled RCT	Aged ≥40 y with T2D, HBA_1c_ ≥6.5% to <12.0%, creatinine clearance of ≥60 mL/min, and high risk for CV events	64.0	6422 (37.4)	Dapagliflozin	Placebo	50.4	Reported as adverse event (dementia or Alzheimer, Lewy body, or vascular dementia)
DAPA-CKD,^30^ 2020	4304	Quadruple-blind, placebo-controlled RCT	Aged ≥18 y with eGFR ≥25 to ≤75 mL/min/1.73 m^2^ at visit 1, evidence of increased albuminuria ≥3 mo before visit 1, and uACR ≥200 to ≤5000 mg/g at visit 1	61.9	1425 (33.1)	Dapagliflozin	Placebo	28.8	Reported as adverse event (dementia)
EMPEROR- Reduced,^31^ 2020	3730	Double-blind, placebo-controlled RCT	Aged ≥18 y with chronic HF (NYHA class II-IV) and reduced ejection fraction, LVEF ≤40%, and elevated NT-proBNP levels	66.9	893 (23.9)	Empagliflozin	Placebo	16	Reported as adverse event (dementia, vascular dementia)
VERTIS CV,^32^ 2020	8246	Double-blind, placebo-controlled RCT	Aged ≥40 y with T2D, HbA_1c_ at start of study between 7.0%-10.5%, BMI ≥18.0, with evidence or a history of atherosclerosis	64.4	2477 (30.0)	Ertugliflozin	Placebo	42	Reported as adverse event (vascular, mixed, or Alzheimer dementia)
EMPEROR- Preserved,^33^ 2021	5988	Double-blind, placebo-controlled RCT	Aged ≥18 y with chronic HF (NYHA class II-IV) and preserved ejection fraction, LVEF >40%, elevated NT-proBNP levels, and structural heart disease within 6 mo prior to visit 1	71.9	2676 (44.7)	Empagliflozin	Placebo	26.2	Reported as adverse event (dementia or Alzheimer, senile, or vascular dementia)
DELIVER,^34^ 2022	6263	Quadruple-blind, placebo-controlled RCT	Aged ≥40 y with a diagnosis of symptomatic HF (NYHA class II-IV), LVEF >40%, and elevated NT-proBNP levels	71.7	2747 (43.9)	Dapagliflozin	Placebo	27.6	Reported as adverse event (Alzheimer dementia)
EMPA-KIDNEY,^35^ 2023	6609	Double-blind, placebo-controlled RCT	Aged ≥18 y, evidence of chronic kidney disease, with an eGFR ≥45 to <90 mL/min/1.73 m^2^, with uACR ≥200	63.9	2192 (33.2)	Empagliflozin	Placebo	24	Reported as adverse event (dementia, Alzheimer dementia)
**GLP-1RAs**									
ELIXA,^36^ 2015	6068	Triple-blind, placebo-controlled RCT	Aged ≥30 y with T2D and acute coronary event within 180 d before screening	60.3	1861 (30.6)	Lixisenatide	Placebo	25	Reported as adverse event (cognitive disorder)
SUSTAIN-6,^37^ 2016	3297	Double-blind, placebo-controlled RCT	Aged ≥50 y with T2D and glycated hemoglobin level ≥7%; eligible if they had not been treated with an antihyperglycemic drug or had been treated with no more than 2 oral antihyperglycemic agents	64.6	1295 (39.3)	Semaglutide	Placebo	25.2	Dementia diagnosis (*ICD-10* codes from routine clinical practice) and use of relevant medication^10^
LEADER,^38^ 2016	9340	Double-blind, placebo-controlled RCT	Aged ≥50 y with T2D and CVD or other risk factors or aged ≥60 y with other risk factors of CVD with HbA_1c_ ≥7.0%	64.3	3337 (35.7)	Liraglutide	Placebo	45.6	Dementia diagnosis (*ICD-10* codes from routine clinical practice) and use of relevant medication^10^; dementia subtype reported as adverse event
EXSCEL,^39^ 2017	14 752	Triple-blind, placebo-controlled RCT	T2D with HbA_1c_ ≥6.5% to ≤10.0%; female patients could not be breastfeeding	62.0	5603 (38.0)	Exenatide	Placebo	38.4	Reported as adverse event (dementia or Alzheimer, senile, mixed, vascular, or frontotemporal dementia)
Harmony,^40^ 2018	9463	Quadruple-blind, placebo-controlled RCT	Aged ≥40 y with T2D and established disease of the coronary, cerebrovascular, or peripheral arterial circulation, with HbA_1c_ >7.0%	64.2	2894 (30.6)	Albiglutide	Placebo	19.2	Reported as adverse event (dementia or Alzheimer or mixed dementia)
REWIND,^41^ 2019	9901	Double-blind, placebo-controlled RCT	Aged ≥50 y with T2D and CV risk factors or previous CV event	66.2	4589 (46.3)	Dulaglutide	Placebo	64.8	Reported as adverse event (dementia or Alzheimer, vascular, or mixed dementia)
PIONEER 6,^42^ 2021	3183	Double-blind, placebo-controlled RCT	Aged ≥50 y with T2D and established CVD or chronic kidney disease or aged ≥60 y with CV risk factors only	66.0	1007 (31.6)	Semaglutide	Placebo	15.9	Reported as adverse event (vascular or Lewy body dementia)
AMPLITUDE-O,^43^ 2021	4076	Quadruple-blind, placebo-controlled RCT	Aged ≥18 y with T2D and HbA_1c_ >7% with CV risk factors or previous CV event, or male, aged ≥50 y or female aged ≥55 y with eGFR ≥25 and <60 mL/min and CV risk factors	64.5	1344 (33.0)	Efpeglenatide	Placebo	21.72	Reported as adverse event (senile dementia)
AMPLITUDE-M,^44^ 2022	406	Quadruple-blind, placebo-controlled RCT	Aged ≥18 y with T2D treated with diet and exercise with HBA_1c_ 7.0%-10.0%	58.9	187 (46.1)	Efpeglenatide	Placebo	14	Reported as adverse event (memory impairment)
SELECT,^45^ 2023	17 604	Quadruple-blind, placebo-controlled RCT	Aged ≥45 y with BMI ≥27 and established CVD	61.6	4872 (27.7)	Semaglutide	Placebo	39.8	Reported as adverse event (dementia or Alzheimer, Lewy body, frontotemporal, or vascular dementia)
Exenatide-PD3,^46^ 2024	254	Quadruple-blind, placebo-controlled RCT	Aged 30-80 y, diagnosed with Parkinson disease, with a Hoehn and Yahr scale score ≤2.5 at screening and a Montreal Cognitive Assessment score of ≥24	61.6	88 (34.6)	Exenatide	Placebo	9	Primary outcome not reported
**Pioglitazone**									
NET-PD,^47^ 2015	210	Quadruple-blind, placebo-controlled RCT	Aged ≥30 y with idiopathic Parkinson disease diagnosed within 5 y of enrollment with a Hoehn and Yahr scale score of ≤2	59.5	62 (29.5)	Pioglitazone	Placebo	10.13	Primary outcome not reported
IRIS,^48^ 2016	3876	Triple-blind, placebo-controlled RCT	Aged ≥40 y and had a qualifying ischemic stroke or transient ischemic attack during the 6 mo before randomization	63.5	1338 (34.5)	Pioglitazone	Placebo	57.6	Primary outcome not reported
TOMORROW,^49^ 2021	3494	Quadruple-blind, placebo-controlled RCT	Aged 65-83 y, cognitively normal at baseline, score of ≥25 on the Mini-Mental State Examination	74.0	1921 (55.0)	Pioglitazone	Placebo	30	Reported as mild cognitive impairment due to Alzheimer disease

Abbreviations: BMI, body mass index (calculated as weight in kilograms divided by height in meters squared); CV, cardiovascular; CVD, cardiovascular disease; eGFR, estimated glomerular filtration rate; GLP-1RA, glucagon-like peptide-1 receptor agonist; HbA_1c_, hemoglobin A_1c_; HF, heart failure; HFrEF, heart failure with reduced ejection fraction; *ICD-10*, *International Statistical Classification of Diseases and Related Health Problems 10th Revision*; LVEF, left ventricular ejection fraction; NT-proBNP, N-terminal pro-B-type natriuretic peptide; NYHA, New York Heart Association; RCT, randomized clinical trial; SGLT2i, sodium-glucose cotransporter-2 inhibitor; T2D, type 2 diabetes; uACR, urine albumin-creatinine ratio.

We tested for heterogeneity between drug classes (GLP-1RAs vs SGLT2is) by dividing the difference in log relative risk by its standard error.^52^ A priori subgroup sensitivity analyses that assessed pooled estimates for trials that reported follow-up above and below the median number of months of follow-up, and including only trials with low risk of bias, was performed. *P* values were 2-sided with a significance threshold of <.05.

## Results

The systematic search of articles published before June 11, 2024, identified 3831 articles. After title and abstract screening, 505 articles were considered potentially relevant. Following full-text and ClinicalTrials.gov review, 26 trials were included (eFigure 1 in Supplement 1). Twenty-three studies reported the incidence of a composite of dementia or cognitive impairment on follow-up and were included in the primary meta-analysis. Three studies were included for the secondary outcome of change in cognitive score only.

### Study Characteristics

Overall, 164 531 participants were included from 26 trials, with a mean (SD) age of 64.4 (3.5) years and 34.9% were women. The mean duration of follow-up was 31.4 (range, 10.1-73.2) months (Table). The publication years ranged from 2015 to 2024; 24 trials were conducted in international sites^25,26,27,28,29,30,31,32,33,34,35,36,38,39,40,41,42,43,44,45,48^ and 2 were conducted in North America.^46,47^ All included trials were placebo controlled (Table).

### Risk of Bias

Risk of bias was assessed for all included trials (eFigure 2 in Supplement 1). The overall risk of bias was categorized as low for all trials. The measurement of the outcome domain was deemed to be low risk for all studies. While there was limited information regarding method of outcome ascertainment, participants and investigators of all included trials were blinded to the intervention allocated. There was no evidence of publication bias for the primary outcome (eFigure 3 in Supplement 1).

### Association of Cardioprotective Glucose-Lowering Therapy With Cognitive Impairment or Dementia

**Figure 1.**
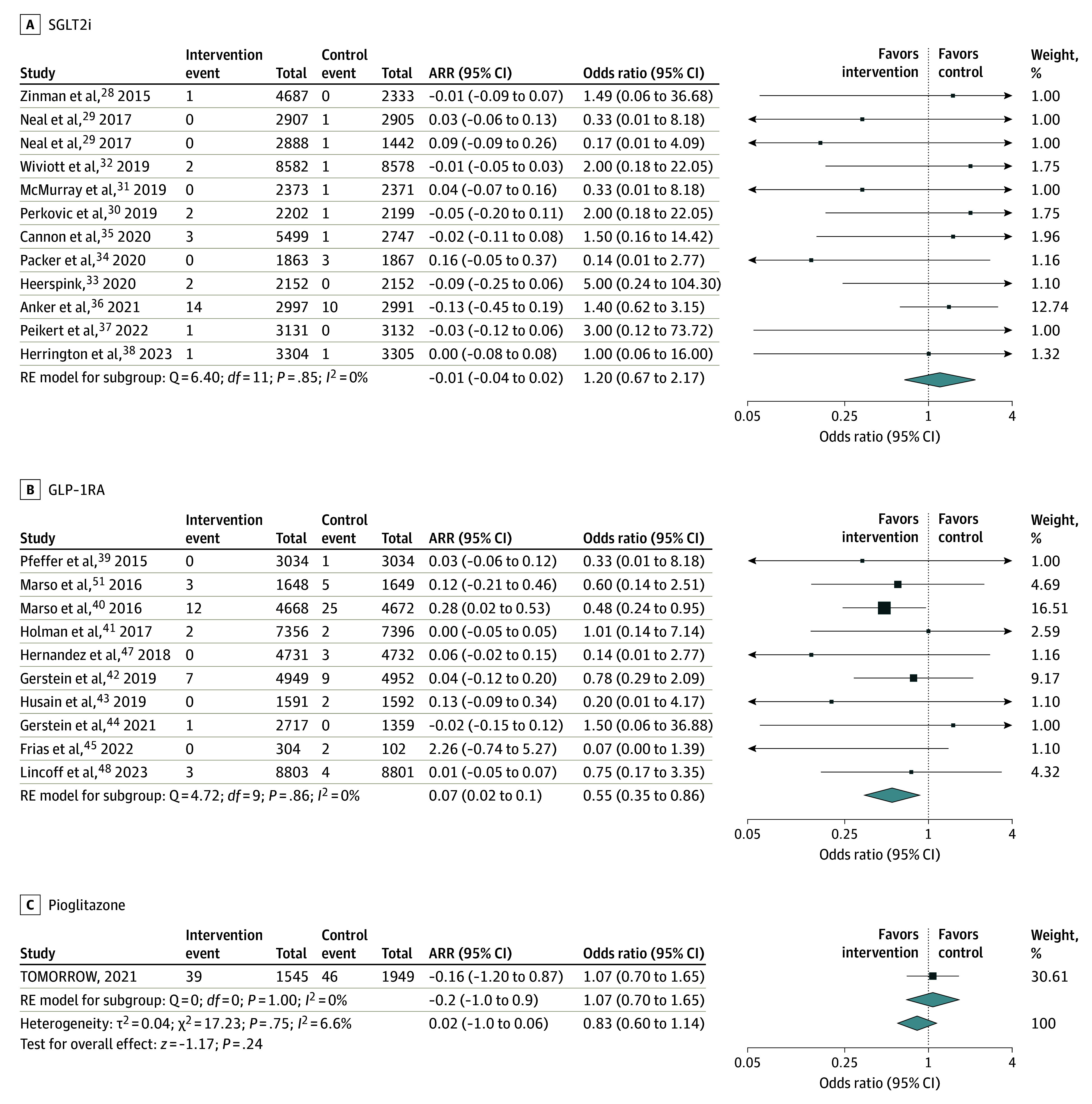
Association of Glucose-Lowering Therapy With All-Cause Dementia Squares and bars represent the mean values and 95% CIs of the effect sizes, while the size of the squares reflects the weight of the study. Diamonds represent the combined effects and the vertical dotted lines, the lines of no effect. Arrows indicate that the values are outside the range of the x-axis. All-cause dementia events extracted from the Nørgaard et al^10^ meta-analysis (Table). ARR indicates absolute risk reduction; RE, random effects.

Twenty-three trials reported dementia or cognitive impairment on follow-up (160 191 participants), 12 trials evaluated SGLT2is, 10 trials evaluated GLP-1RAs, and 1 trial evaluated pioglitazone (no trials of metformin were identified).^25,26,27,28,29,30,31,32,33,34,35,36,37,38,39,41,42,43,44,45^ Dementia or cognitive impairment was diagnosed in 93 participants in the intervention group and 119 participants in the control group on follow-up. Glucose-lowering therapy was not significantly associated with a reduction in cognitive impairment or dementia (0.12% vs 0.14% over a mean follow-up of 31.8 months; OR, 0.83 [95% CI, 0.60-1.14]; ARR, 0.02% [95% CI, −1.00% to 0.09%]; *I^2^* = 6.6%). Glucose-lowering therapy with GLP-1RAs (OR, 0.55 [95% CI, 0.35-0.86]), but not SGLT2is (OR, 1.20 [95% CI, 0.67-2.17]), was statistically significantly associated with a reduction in cognitive impairment or dementia (*P* value for heterogeneity = .04; Figure 1). Meta-regression analysis showed no significant association of the proportion of women enrolled with all-cause dementia (*P* = .08) (eFigure 4 in Supplement 1).

### Association of Glucose-Lowering Therapy With Dementia Subtypes

**Figure 2.**
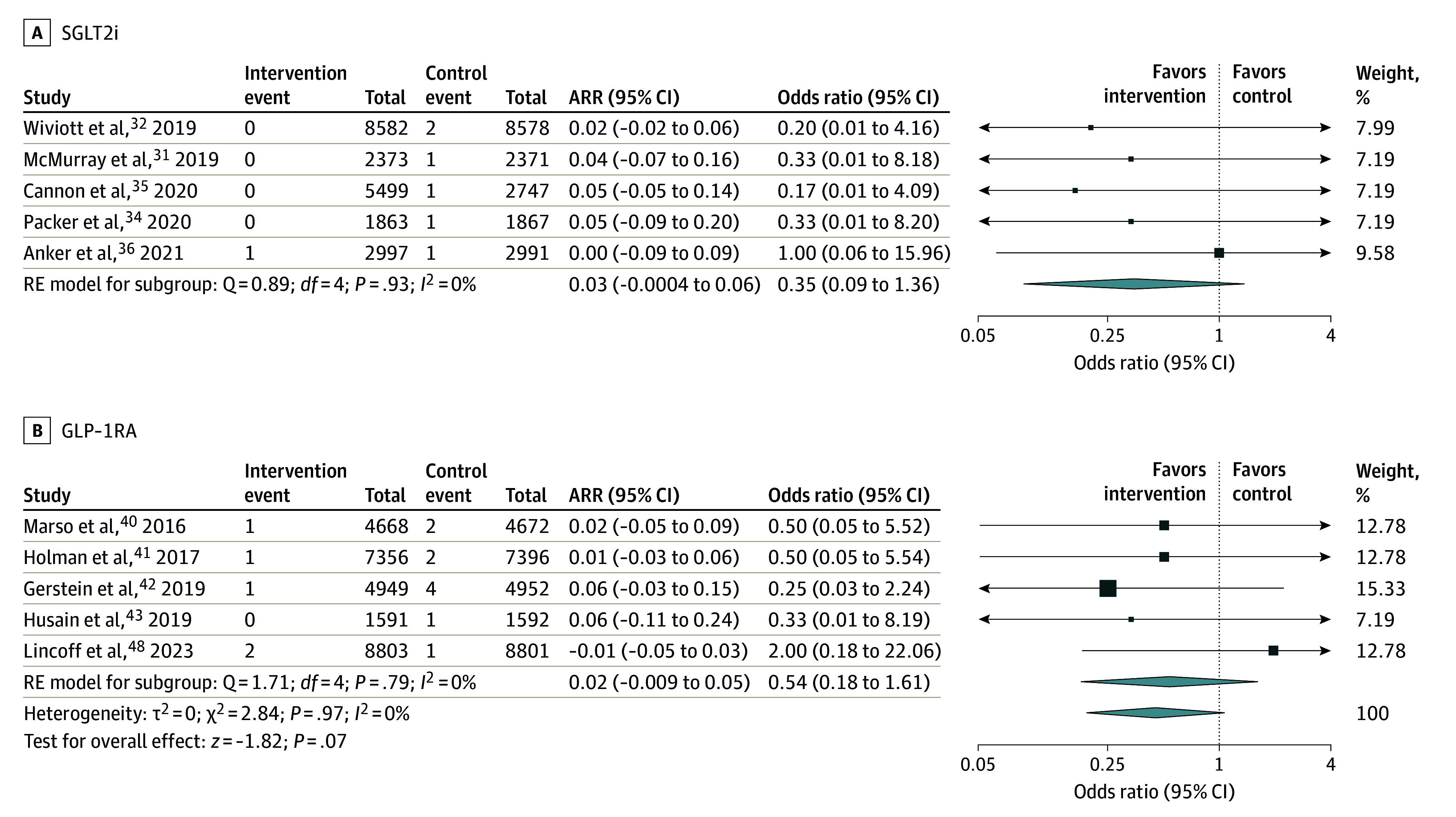
Association of Glucose-Lowering Therapy With Vascular Dementia Squares and bars represent the mean values and 95% CIs of the effect sizes, while the size of the squares reflects the weight of the study. Arrows indicate that the values are outside the range of the x-axis. Diamonds represent the combined effects and the vertical dotted lines, the lines of no effect. ARR indicates absolute risk reduction; RE, random effects.

**Figure 3.**
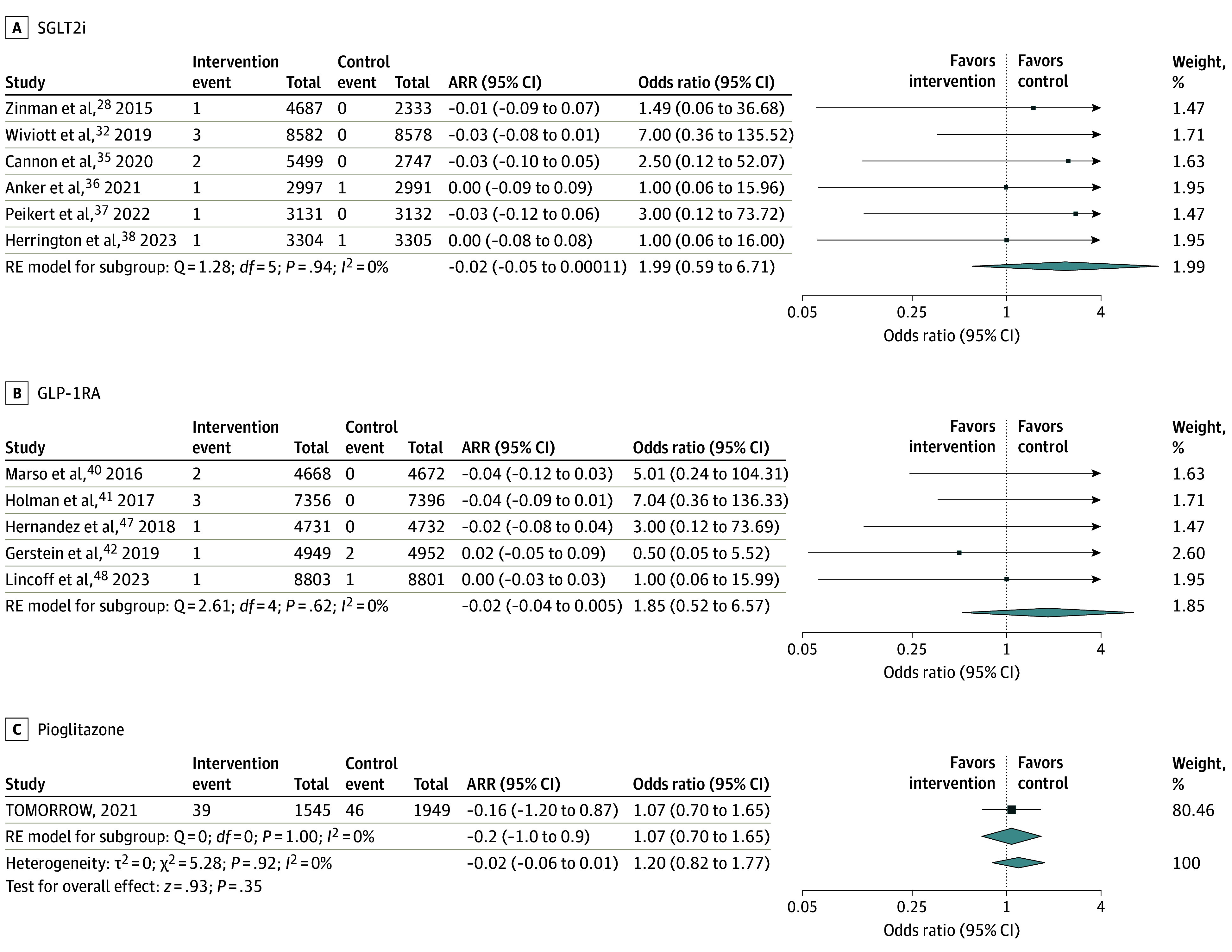
Association of Glucose-Lowering Therapy With Alzheimer Dementia Squares and bars represent the mean values and 95% CIs of the effect sizes, while the size of the squares reflects the weight of the study. Arrows indicate that the values are outside the range of the x-axis. Diamonds represent the combined effects and the vertical dotted lines, the lines of no effect. ARR indicates absolute risk reduction; RE, random effects.

Dementia subtypes included vascular dementia, Alzheimer dementia, Lewy body dementia, and frontotemporal dementia. Ten trials reported rates of vascular dementia on follow-up (94 648 participants).^28,29,31,32,33,38,39,41,42,45^ Vascular dementia was diagnosed in 6 participants in the intervention group and 16 participants in the control group on follow-up. Glucose-lowering therapy was not significantly associated with a reduction in vascular dementia (0.01% vs 0.03% over a mean follow-up of 35.7 months; OR, 0.45 [95% CI, 0.19-1.07]; *I^2^* = 0.0%). This was consistent across drug classes (SGLT2i OR, 0.35 [95% CI, 0.09-1.36]; GLP-1RA OR, 0.38 [95% CI, 0.18-1.61]; *P* value for heterogeneity = .93; Figure 2). Twelve trials reported rates of Alzheimer dementia (115 840 participants).^25,29,32,33,34,35,38,39,40,41,45,49^ Alzheimer dementia was diagnosed in 56 participants in the intervention group and 51 participants in the control group on follow-up. Glucose-lowering therapy was not associated with a significant reduction in Alzheimer dementia (0.09% vs 0.09% over a mean follow-up of 37.1 months; OR, 1.20 [95% CI, 0.82-1.77]; *I^2^* = 0.0%). This was consistent across drug classes (SGLT2i OR, 1.99 [95% CI, 0.59-6.71]; GLP-1RA OR, 1.85 [95% CI, 0.52-6.57]; pioglitazone OR, 1.07 [95% CI, 0.70-1.65]; Figure 3). Four trials reported rates of Lewy body dementia (47 287 participants).^29,38,42,45^ Lewy body dementia was diagnosed in 1 participant in the intervention group and 3 participants in the control group on follow-up. Glucose-lowering therapy was not associated with a significant reduction in Lewy body dementia (0.004% vs 0.01% over a mean follow-up of 37.9 months; OR, 0.58 [95% CI, 0.12-2.86]; *I^2^* = 0.0%) (eFigure 5 in Supplement 1). There were insufficient data to report a meta-analytic estimate for the frontotemporal dementia subtype (1 trial).^39^

### Association of Glucose-Lowering Therapy With Change in Cognitive Score

Three trials reported on change in cognitive score.^46,47,48^ McGarry et al reported no significant difference in cognition, measured by Scales for Outcomes in Parkinson’s Disease Cognition, between exenatide and placebo over a mean follow-up of 36 months.^46^ The Pioglitazone in Early Parkinson’s Disease trial reported no difference in cognition, measured by Mattis Dementia Rating Scale scores, between the pioglitazone and placebo groups over a mean follow-up of 10.1 months.^47^ The Insulin Resistance Intervention After Stroke trial reported no significant difference in cognition, measured by Modified Mini-Mental State Examination, associated with pioglitazone compared with placebo over a mean follow-up of 57.6 months.^48^

A priori subgroup sensitivity analyses for the primary outcome assessing pooled estimates for trials that reported years of follow-up above and below the median follow-up duration did not materially alter findings (eFigure 6 in Supplement 1).

## Discussion

This meta-analysis, which included 23 trials with 160 191 participants for the primary outcome analysis, did not report a significant reduction in dementia or cognitive impairment when all drug classes were considered. However, glucose-lowering therapy with GLP-1RAs, but not SGLT2is or pioglitazone, was associated with a significantly lower risk of dementia or cognitive impairment, compared with controls. Glucose-lowering therapy was not associated with a significant reduction in vascular or Alzheimer dementia. Vascular dementia event rates were numerically lower (not statistically significant) in the glucose-lowering group compared with controls. In contrast, Alzheimer dementia event rates were numerically higher (not statistically significant) in the glucose-lowering group compared with controls.

These findings add new information to existing literature. A 2023 meta-analysis of randomized clinical trials did not report a significant reduction in dementia, and included 21 randomized clinical trials, including DPP-4 inhibitors, GLP1-RAs, and SGLT2is.^8^ This meta-analysis differed in that the analysis was confined to glucose-lowering therapy with proven effectiveness in reducing the risk of cardiovascular disease, and included additional trials for GLP1-RA and SGLT2i classes. A selected pooled analysis of 3 randomized clinical trials and a nationwide Danish registry-based cohort reported that GLP-1RA exposure was associated with reduced risk of dementia.^10^ These findings extend the findings of this study by including additional trials evaluating GLP-1RAs identified through a systematic search, and also including other drug classes that have demonstrated cardiovascular benefit, which allowed treatment effects to be determined among drug classes.

Diabetes is associated with an increased risk of dementia, expected to be primarily mediated through vascular injury, but may also increase brain atrophy through other mechanisms.^53^ The study hypothesis was that glucose-lowering medications associated with significant reductions in cardiovascular events would also reduce the risk of dementia, acknowledging that, in general, the magnitude of association of common vascular risk factors with dementia is lower than risk reported for cardiovascular events. For example, antihypertensive therapy is associated with a 27% relative risk reduction in stroke,^54^ but a 7% relative risk reduction in dementia.^23^ As a result, large sample sizes with extended periods of follow-up are required to detect a significant reduction in dementia risk. Animal studies suggest that GLP-1RAs may reduce dementia risk through a number of potential mechanisms, in addition to neuroprotection mediated through reduced cardiovascular risk (eg, through anti-inflammatory effects on neuronal structures, antioxidative effects, and reduction in neuronal apoptosis).^55^ Similarly, SGLT2is have demonstrated neuroinflammatory and antioxidative effects in addition to cardiovascular prevention.^56^

In this meta-analysis, a larger risk reduction in dementia was associated with randomization to receive GLP-1RAs than SGLT2i classes. This may be partly explained by differences in populations enrolled, with a higher all-cause dementia event rate noted among the control group of GLP-1RA trials compared with SGLT2i trials (0.14% vs 0.05%), with a resultant increase in statistical power to detect associations. Indirect comparison also suggests that GLP-1RAs may have a larger magnitude of effect on cardiovascular risk than SGLT2is.^16,57^ While none of the eligible clinical trials included a specific population with cognitive impairment, findings may have implications for choice of glucose-lowering therapy in patients with diabetes and higher risk of dementia. However, large randomized clinical trials should be conducted that are dedicated to addressing optimal glucose-lowering therapy in patients with cognitive impairment. It is plausible that the efficacy of glucose-lowering therapies for dementia outcomes may differ by sex and APOE4 status due to differences in glucose metabolism.^58,59^ Future studies reporting dementia outcomes by APOE4 status and sex are required to evaluate this.

A limitation of this meta-analysis is that the majority of clinical trials did not systematically evaluate participants for dementia, resulting in a low event rate. Other possible reasons for the low event rate include mean age of trial participants and relatively short duration of follow-up. However, as all clinical trials were placebo controlled, the relative risk estimates are expected to be unbiased. The incidence of all-cause dementia and vascular dementia among adults with diabetes in prospective cohort studies was estimated to be 14.2 to 16.8 per 1000 patient-years and 1.8 to 3.2 per 1000 patient-years, respectively, more than double the rate reported in this study.^60,61^ Therefore, the absolute risk reduction reported is most likely an underestimate, and the real-world absolute risk reduction of cardioprotective glucose-lowering therapy is likely to be greater by approximately 0.5% to 0.6%.

A number of randomized clinical trials evaluating the neuroprotective effects of GLP1-RAs and SGLT2is are in progress. Studies are ongoing among populations with early Alzheimer disease or mild cognitive impairment, such as EVOKE (NCT04777396)^62^ and LIGHT-MCI (NCT05313529), and healthy volunteers with vascular risk factors, such as OxSENSE (NCT06363487).

### Limitations

This study has limitations. First, there are inherent challenges in performing and interpreting a meta-analysis with heterogenous interventions, and definitions of the outcomes of dementia. The study reported meta-analytic estimates by drug classes. There were no randomized clinical trials identified evaluating metformin compared with controls that reported dementia outcomes. Second, dementia outcomes were not ascertained as an outcome of interest; rather, they were reported as adverse events. Bias due to outcome misclassification may have arisen due to incomplete ascertainment of dementia diagnoses. However, as investigators and participants were blinded to treatment allocation, this should not affect the validity of relative comparisons, as both groups of the included randomized clinical trials were likely to have been equally affected by outcome misclassification and underreporting. Third, the low event rates, which are likely related to the average age of the study population, limited duration of follow-up and method of ascertainment (as adverse events rather than systematically sought), reducing power to detect differences in treatment effect. This is a substantial limitation of this study, and further research evaluating the efficacy of glucose-lowering therapies to reduce risk of dementia outcomes in suitably designed trials (ie, with long duration of follow-up and population, including adults in middle-late life) is required. It is important to note that event rates for less common dementia subtypes, such as Lewy body dementia, were very low and therefore should be considered exploratory. Fourth, the duration of follow-up of included studies was relatively short (mean follow-up, 31.4 months). Fifth, the study did not report estimates within subgroups of trial populations (eg, by sex), as only summary-level data were available. Sixth, further research, including randomized clinical trials specifically evaluating the effect of glucose-lowering therapy on incident dementia and cognitive outcomes, is needed. While this study reported on the relative association of glucose-lowering therapy, due to underreporting, the absolute effect is likely larger. It is imperative that these trials are of sufficient follow-up duration to ascertain cognitive outcomes.

## Conclusions

In this meta-analysis of randomized clinical trials, glucose-lowering therapy with GLP1-RAs, but not SGLT2is or pioglitazone, was associated with a statistically significant reduction in dementia or cognitive impairment.

## Notes

Supplement 1. eMethods. Search strategy eFigure 1. PRISMA flow eFigure 2. Risk of bias eFigure 3. Funnel plot eFigure 4. Association of glucose-lowering therapy with all dementia, meta-regression by proportion of female participants enrolled eFigure 5. Association of glucose-lowering therapy with Lewy body Dementia eFigure 6. Subgroup analysis above and below median follow-up

Supplement 2. Data Sharing Statement
