# Supplementary material for: Cardioprotective Glucose-Lowering Agents and Dementia Risk: A Systematic Review and Meta-Analysis
Source: JAMA Neurol. 2025 Apr 7;82(5):450–60. doi: 10.1001/jamaneurol.2025.0360 (PMC11976645; doi:10.1001/jamaneurol.2025.0360)
Supplement: Supplement 1. — eMethods. Search strategy eFigure 1. PRISMA flow eFigure 2. Risk of bias eFigure 3. Funnel plot eFigure 4. Association of glucose-lowering therapy with all dementia, meta-regression by proportion of female participants enrolled eFigure 5. Association of glucose-lowering therapy with Lewy body Dementia eFigure 6. Subgroup analysis above and below median follow-up [file jamaneurol-e250360-s001.pdf]

Supplemental Online Content

Seminer A, Mulihano A, O'Brien C, et al. Cardioprotective glucose-lowering agents and dementia risk: a systematic review and meta-analysis. *JAMA Neurol.* Published online April 7, 2025. doi:10.1001/jamaneurol.2025.0360

eMethods. Search strategy
eFigure 1. PRISMA flow
eFigure 2. Risk of bias
eFigure 3. Funnel plot
eFigure 4. Association of glucose-lowering therapy with all dementia, meta-regression by proportion of female participants enrolled
eFigure 5. Association of glucose-lowering therapy with Lewy body Dementia
eFigure 6. Subgroup analysis above and below median follow-up

This supplemental material has been provided by the authors to give readers additional information about their work.

© 2025 American Medical Association. All rights reserved, including those for text and data mining, AI training, and similar technologies.

Table of Contents

eMethods – Search strategy 3

Figures 4

eFigure 1 – PRISMA flow 4

eFigure 2 – Risk of bias 5

eFigure 3 – Funnel plot 6

eFigure 4 – Association of glucose-lowering therapy with all dementia, meta-regression by proportion of female participants enrolled 7

eFigure 5 – Association of glucose-lowering therapy with Lewy body Dementia 8

eFigure 6 – Subgroup analysis above and below median follow-up 9

© 2025 American Medical Association. All rights reserved, including those for text and data mining, AI training, and similar technologies.

eMethods – Search strategy

SGLT-2 OR sodium-glucose cotransporter-2 inhibitor OR sglt-2 inhibitor OR empagliflozin OR canagliflozin OR luseogliflozin OR dapagliflozin OR ertugliflozin OR tofogliflozin OR sergliflozin OR remogliflozin OR ipragliflozin

OR

Glp-1 OR glucagon-like peptide-1 receptor OR glucagon-like peptide-1 receptor agonist OR lixisenatide OR liraglutide OR semaglutide OR albiglutide OR dulaglutide OR exanatide

OR

biguanide OR metformin

OR

pioglitazone

AND

Usual care OR placebo

AND

RCT sensitive search strategy

Note – dementia/cognitive impairment terms are intentionally omitted as they are unlikely to be included in study abstracts.

© 2025 American Medical Association. All rights reserved, including those for text and data mining, AI training, and similar technologies.

Figures

eFigure 1 – PRISMA flow

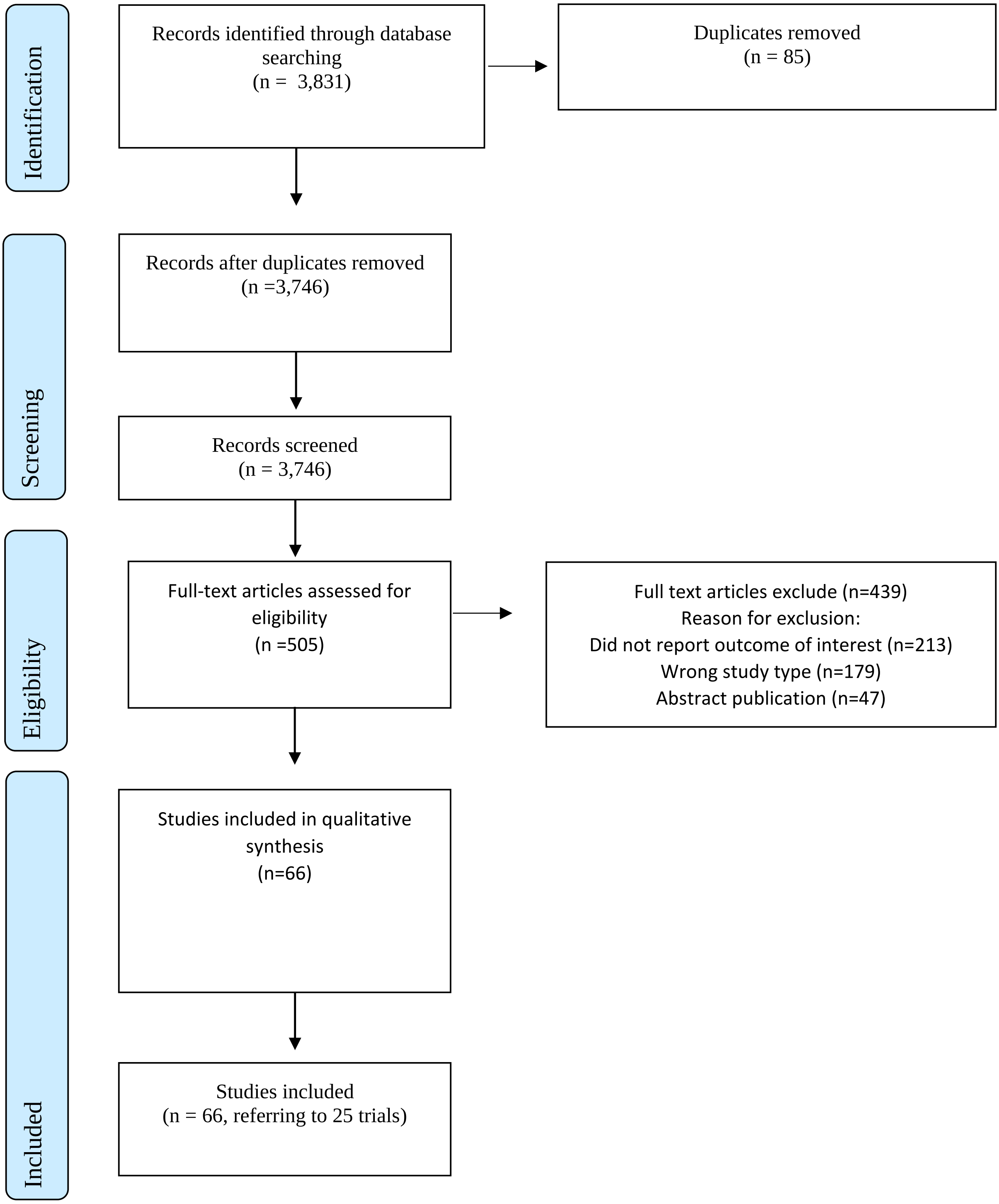

© 2025 American Medical Association. All rights reserved, including those for text and data mining, AI training, and similar technologies.

eFigure 2 – Risk of bias

Unique ID	Experimental	Comparator	D1	D2	D3	D4	D5	Overall
EMPA-REG OUTC	Empagliflozin	Placebo	+	+	+	+	+	+
CANVAS-R	Canagliflozin	Placebo	+	+	+	+	+	+
CREDENCE	Canagliflozin	Placebo	+	+	+	+	+	+
DAPA-HP	Dapagliflozin	Placebo	+	+	+	+	+	+
DECLARE-TIMI 5	Dapagliflozin	Placebo	+	+	+	+	+	+
DAPA-CKD	Dapagliflozin	Placebo	+	+	+	+	+	+
EMPEROR-Redu	Empagliflozin	Placebo	+	+	+	+	+	+
VERTIS CV	Ertugliflozin	Placebo	+	+	+	+	+	+
EMPEROR-Prese	Empagliflozin	Placebo	+	+	+	+	+	+
CANVAS	Canagliflozin	Placebo	+	+	+	+	+	+
DELIVER	Dapagliflozin	Placebo	+	+	+	+	+	+
EMPA-KIDNEY	Empagliflozin	Placebo	+	+	+	+	+	+
ELIXA	Lixisenatide	Placebo	+	+	+	+	+	+
LEADER	Liraglutide	Placebo	+	+	+	+	+	+
EXSCEL	Exenatide	Placebo	+	+	+	+	+	+
HARMONY	Albiglutide	Placebo	+	+	+	+	+	+
REWIND	Dulaglutide	Placebo	+	+	+	+	+	+
PIONEER-6	Semaglutide	Placebo	+	+	+	+	+	+
AMPLITUDE-O	Efpeglenatide	Placebo	+	+	+	+	+	+
AMPLITUDE-M	Efpeglenatide	Placebo	+	+	+	+	+	+
SUSTAIN	Semaglutide	Placebo	+	+	+	+	+	+
TOMORROW	Pioglitazone	Placebo	+	+	+	+	+	+
SELECT	Semaglutide	Placebo	+	+	+	+	+	+

+ Low risk
! Some concerns
- High risk

D1 Randomisation process
D2 Deviations from the intended interventions
D3 Missing outcome data
D4 Measurement of the outcome
D5 Selection of the reported result

© 2025 American Medical Association. All rights reserved, including those for text and data mining, AI training, and similar technologies.

eFigure 3 – Funnel plot

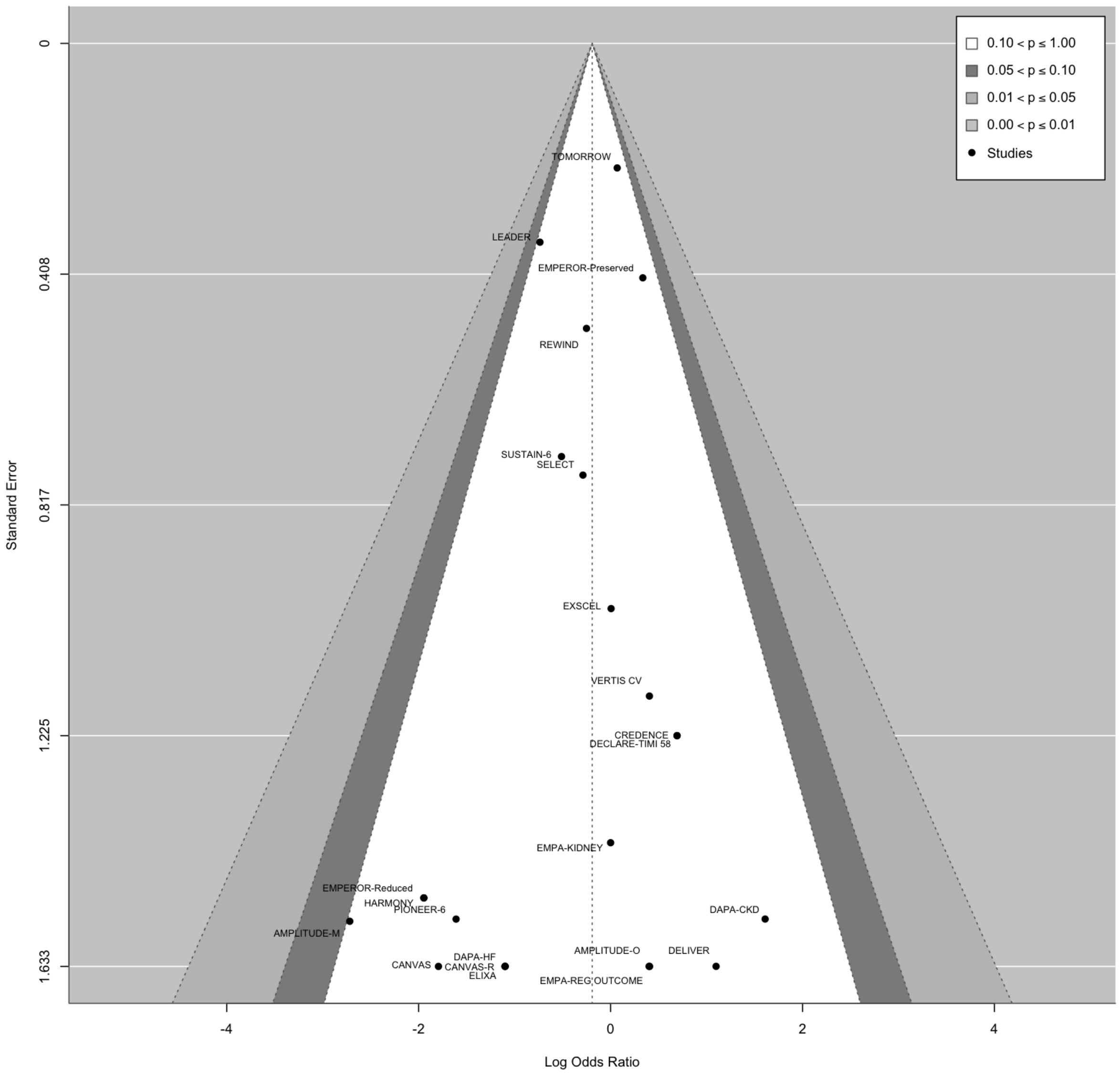

eFigure 3 – Contour enhanced funnel plot for included trials, primary outcome (dementia or cognitive impairment). Different levels of statistical significance for studies are indicated by the shaded regions, detailed within the figure. The grey vertical line represents the summary estimate. Each point is labelled to identify corresponding study/studies with label offset to the left of point.

© 2025 American Medical Association. All rights reserved, including those for text and data mining, AI training, and similar technologies.

eFigure 4 – Association of glucose-lowering therapy with all dementia, meta-regression by proportion of female participants enrolled

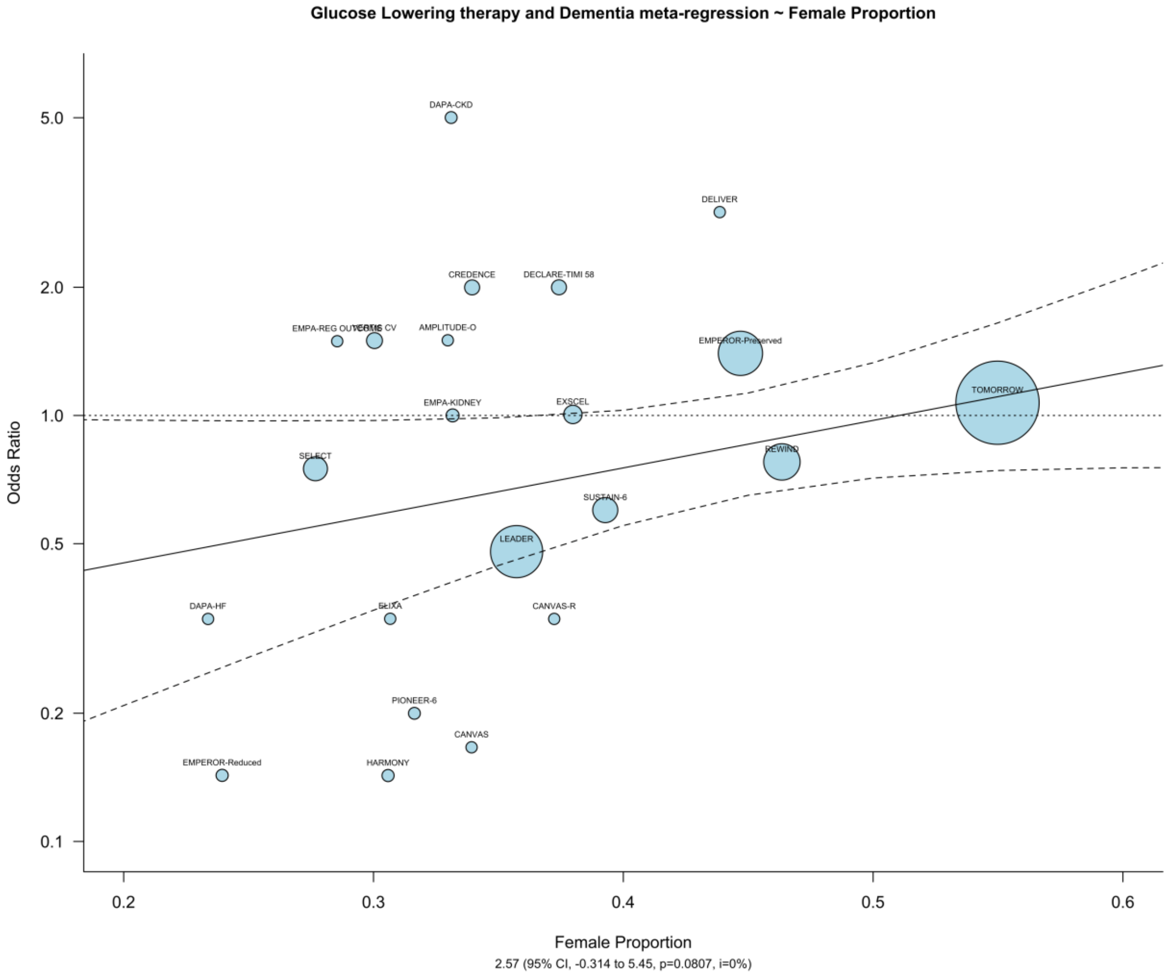

© 2025 American Medical Association. All rights reserved, including those for text and data mining, AI training, and similar technologies.

eFigure 5 – Association of glucose-lowering therapy with Lewy body Dementia

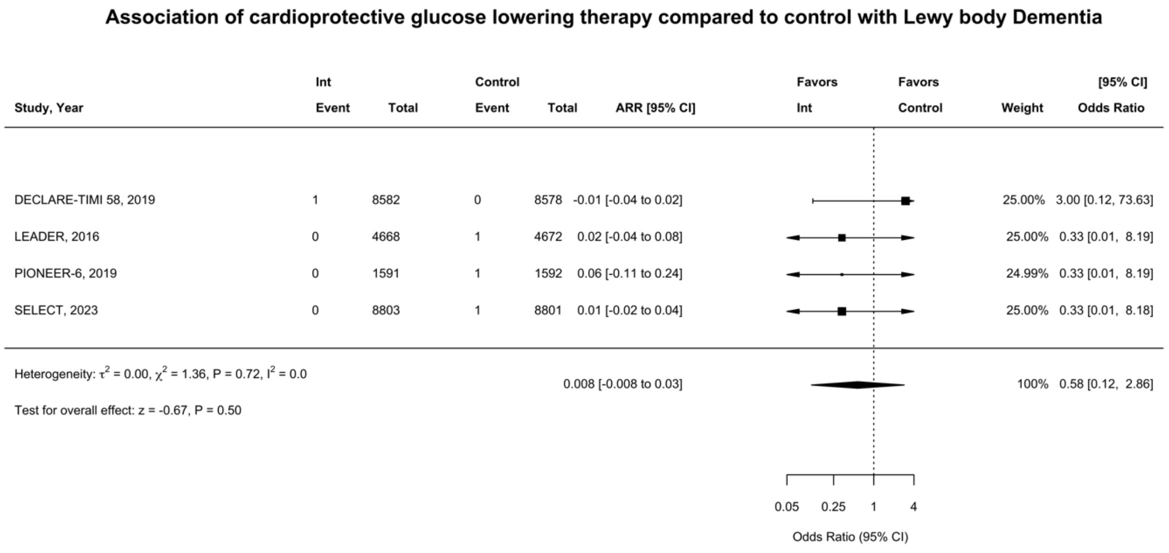

eFigure 5 – Forest plot demonstrating the association of glucose lowering therapy compared to control with Lewy body dementia. The squares and bars represent the mean values and 95% confidence intervals of the effect sizes, while the area of the squares reflects the weight of the studies. The combined effects appear as diamonds and the vertical dashed line represents the line of no effect.
Int - Intervention, CI-Confidence Interval

© 2025 American Medical Association. All rights reserved, including those for text and data mining, AI training, and similar technologies.

eFigure 6 – Subgroup analysis above and below median follow-up

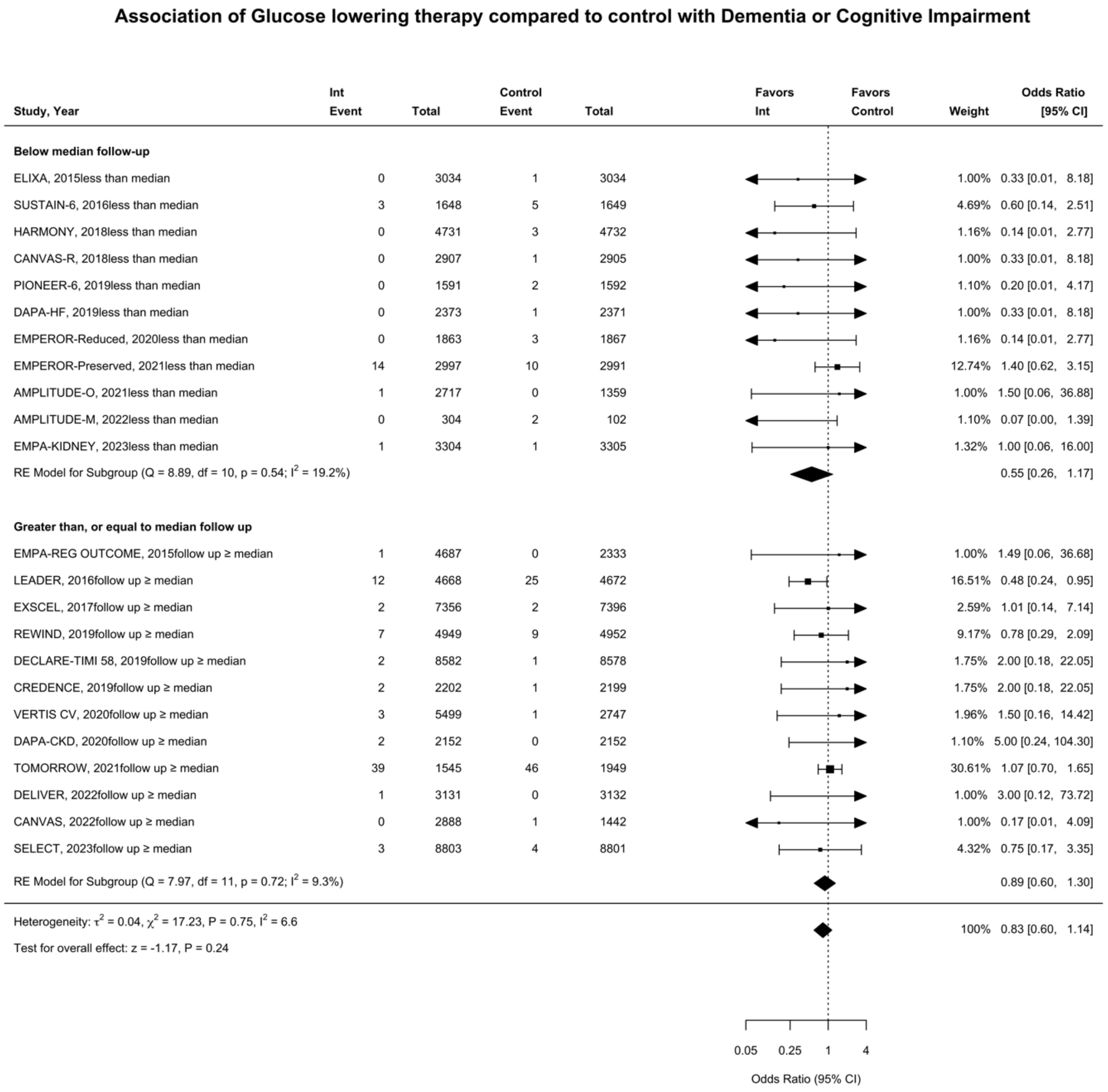

eFigure 6 – Forest plot demonstrating the association of glucose lowering therapy compared to control with dementia or cognitive impairment by follow up duration. The squares and bars represent the mean values and 95% confidence intervals of the effect sizes, while the area of the squares reflects the weight of the studies. The combined effects appear as diamonds and the vertical dashed line represents the line of no effect.
Int - Intervention, CI-Confidence Interval

© 2025 American Medical Association. All rights reserved, including those for text and data mining, AI training, and similar technologies.
