# Supplementary material for: Cardioprotective Glucose-Lowering Agents and Dementia Risk: A Systematic Review and Meta-Analysis
Source: JAMA Neurol. 2025 Apr 7;82(5):450–60. doi: 10.1001/jamaneurol.2025.0360 (PMC11976645; doi:10.1001/jamaneurol.2025.0360)
Supplement: Supplement 2. — Data Sharing Statement [file jamaneurol-e250360-s002.pdf]

Data Sharing Statement

Seminer. Cardioprotective Glucose-Lowering Agents and Dementia Risk. *JAMA Neurol*. Published April 07, 2025. doi:10.1001/jamaneurol.2025.0360

Data
Data available: No

Additional Information
Explanation for why data not available: The data that support the findings of this study are available from the corresponding author upon reasonable request.
